# Advancing the environmental DNA and RNA toolkit for aquatic ecosystem monitoring and management

**DOI:** 10.7717/peerj.19119

**Published:** 2025-03-18

**Authors:** Xavier Pochon, Holly A. Bowers, Anastasija Zaiko, Susanna A. Wood

**Affiliations:** 1 Molecular Surveillance Team, Cawthron Institute, Nelson, Tasman, New Zealand; 2 Institute of Marine Science, University of Auckland, Auckland, New Zealand; 3 Moss Landing Marine Labs, San Jose State University, San Jose, United States of America; 4 Sequench Ltd., Nelson, New Zealand; 5 Lincoln University, Christchurch, New Zealand

**Keywords:** eDNA, eRNA, Biosurveillance, Aquaculture, Aquatic ecosystems, Biodiversity, Biotic index, Invasive species, Environmental management, Metabarcoding

## Abstract

The application of environmental DNA (eDNA) and RNA (eRNA) technologies to aquatic ecosystem monitoring and management has increased rapidly in the last decade. These methods are providing many new and exciting opportunities for enhanced biodiversity assessment, ecological health evaluation, and species detection. This special issue of PeerJ Life and Environment brings together 20 innovative studies that collectively advance the eDNA toolkit. Four key themes are covered: (i) Methodological advancements, (ii) Ecological health assessments and biomonitoring, (iii) Species detection, and (iv) Application and management. The studies cover a suite of topics including; optimizing sample collection, developing species-specific assays, evaluating bioindicator species, assessing microbial activity, and biodiversity monitoring in diverse freshwater and marine habitats. Emerging applications, such as the use of genome skimming to identify new fish markers, showcase the many new advancements in this field. The studies in this issue also highlight challenges, including the need for standardized protocols and ethical considerations that must be addressed before these tools can be implemented or adopted for decision making at national or global scales. Together, these contributions demonstrate the transformative potential of environmental nucleic acids’ technologies for advancing aquatic conservation and management. By bridging methodological rigor with applied research, the studies in this special issue provide an important resource for researchers, policymakers, and practitioners committed to sustainable aquatic ecosystem stewardship.

## Introduction

Aquatic ecosystems are among the most biodiverse and ecologically significant environments on Earth, providing essential services such as carbon sequestration, nutrient cycling, and habitat provision (Dudgeon et al., 2006; Barbier et al., 2011). However, these ecosystems are increasingly threatened by natural and anthropogenic pressures, including climate change, pollution, habitat destruction, and the introduction of invasive species (Smith, 2003; Du Plessis, 2022). Effective monitoring and management strategies are urgently needed to safeguard their health. Traditional monitoring methods, while valuable, often face limitations in terms of cost, labor intensity, and the ability to capture comprehensive biodiversity data, particularly for cryptic or rare species. Molecular approaches such as environmental DNA (eDNA) and RNA (eRNA) have emerged as powerful tools for advancing aquatic ecosystem monitoring and management (Taberlet et al., 2012).

Environmental DNA refers to genetic material shed by organisms into their environment through processes such as excretion, reproduction, and decay. Environmental RNA provides insights into the active biological processes and metabolic states of organisms, thereby offering complementary information to eDNA. Together, these molecular tools enable non-invasive, high-resolution assessments of biodiversity, ecological health, and species distributions across diverse aquatic habitats (Thomsen & Willerslev, 2015).

The rapid development of DNA technologies has been driven by advances in high-throughput sequencing, bioinformatics, and molecular assay design. These innovations have expanded the scope and scale of molecular monitoring, allowing researchers to detect and quantify organisms with unprecedented precision. For instance, eDNA metabarcoding enables the simultaneous identification of multiple species from a single environmental sample, providing a cost-effective and scalable approach to biodiversity monitoring. Similarly, eRNA analyses offer insights into the functional activity of microbial communities or the living component in aquatic biodiversity assessment (Pochon et al., 2017; Cristescu, 2019).

Despite their transformative potential, the implementation of environmental nucleic acids in aquatic ecosystem monitoring is not without challenges. Methodological issues such as representative sampling, sample preservation, inhibitor removal, and taxonomic resolution remain areas of active research. Additionally, the integration of molecular data into existing monitoring or policy frameworks requires interdisciplinary collaboration and stakeholder engagement to ensure their relevance and applicability to management goals. Ethical considerations, including data ownership, privacy, and the potential misuse of molecular information, also warrant careful attention.

This special issue of *PeerJ Life and Environment* brings together 20 cutting-edge studies that address these challenges and highlight the diverse applications of eDNA technologies. Notably, none of the studies in this special issue specifically investigated eRNA, highlighting the need for further research to develop and apply eRNA-based methodologies in aquatic ecosystem monitoring. The contributions span four key themes: (i) Methodological advancements, (ii) Ecological health assessments and biomonitoring, (iii) Species detection, and (iv) Application and management. Collectively, they demonstrate the versatility and impact of molecular tools in advancing our understanding and management of aquatic ecosystems. By collating these contributions, this special issue aims to inspire further innovation and collaboration in the field of molecular monitoring. It underscores the importance of standardization, technological integration, and interdisciplinary approaches to fully harness the potential of eDNA tools.

## Special Issue Themes

Nine studies focused on methodological advancements, ranging from innovative eDNA filtration systems, new species-specific and microfluidic assays, genome skimming for new fish markers development, through to systematic comparisons of traditional and eDNA-based biodiversity measurements (Chen et al., 2022; Hoban et al., 2022; Willassen et al., 2022; DeHart et al., 2023; Hauck et al., 2023; Hong et al., 2023; Picard et al., 2023; Quebedeaux et al., 2023; Holmes et al., 2024). Five studies focused on ecological health assessment and biomonitoring, including the development of biodiversity metrics using metabarcoding of bacterial and/or eukaryotic assemblages for assessing river health, the examination of microbial dynamics of aquaculture shrimp larvae, and the use of metabarcoding for evaluating the impacts of a mining disaster on estuarine meiofaunal assemblages (Aunins et al., 2023; Callac et al., 2023; Coppo et al., 2023; Chavarria, Batista & Saltonstall, 2024; Wilkinson et al., 2024). Three studies focused on species detection, developing species-specific quantitative PCR (qPCR) or droplet digital PCR (ddPCR) assays for detecting the Asian paddle crab, the common musk turtle, and a range of fish during spawning aggregations (Feng & Lougheed, 2023; Gonzalez Colmenares et al., 2023; Simpson et al., 2023). Finally, three studies showcased applications and management, spanning the application of supervised machine learning of metabarcoding data for assessing benthic environmental quality at coastal marine aquaculture sites, through to a valuable review of 60 marine eDNA metabarcoding studies that identifies barriers to data usability and accessibility and a guide outlining five key steps for implementing eDNA metabarcoding in marine ecosystems monitoring (Gold et al., 2022; Leontidou, Rubel & Stoeck, 2023; Shea et al., 2023).

## Summary of Contributions

The 20 articles published in this special issue are summarized below, organized by themes:

### Methodological advancements

1. Chen et al. (2022) examined the breeding phenology of the Western chorus frog (*Pseudacris maculata*) using environmental DNA (eDNA) droplet digital PCR (ddPCR) and acoustic monitoring. The results suggest that eDNA detection lagged behind acoustic monitoring by a few days but can effectively complement traditional methods, especially for non-calling amphibians. The study highlighted the importance of managing PCR inhibitors to enhance eDNA quantification and emphasized the utility of eDNA for monitoring cryptic species.

2. Hoban et al. (2022) explored the use of genome skimming for generating comprehensive mitochondrial and nuclear ribosomal DNA barcoding markers for marine fishes. By testing various DNA extraction and shearing methods, the authors successfully produced complete mitogenomes for 11 of 12 species and assembled nuclear ribosomal repeats. The findings indicate that genome skimming is an efficient, cost-effective approach for building reference databases.

3. Willassen et al. (2022) compared eDNA metabarcoding with traditional biodiversity assessment methods in Svalbard fjords. While metabarcoding detected 69 additional species often overlooked by visual sorting, it identified only 20.6% of morphologically identified Polychaeta species. Factors such as uneven DNA distribution, PCR bias, and primer mismatch were identified as the possible causes.

4. DeHart et al. (2023) presented a low-cost ($350) eDNA filtration system designed for field and laboratory use, aimed at standardizing aquatic eDNA collection methods. The system can filter water samples at over 150 mL/min using 0.22 mm and 0.45 mm Sterivex filters, improving eDNA recovery and ensuring sample integrity. The portable system is battery-powered, making it adaptable for various environments. The study emphasized the need for standardized filtration protocols to improve cross-study comparisons and enhance the reproducibility of eDNA research.

5. Hauck et al. (2023) explored the efficacy of eDNA metabarcoding to assess biodiversity in freshwater mussel populations in Alabama, particularly in the Sipsey River. They developed a microfluidic array targeting various aquatic species and compared this with traditional sampling and identification methods. While the eDNA results aligned broadly with the findings from traditional surveys, discrepancies occurred. The study highlighted the challenges of using eDNA in murky river conditions and proposed improvements for freshwater mussel monitoring using molecular techniques.

6. Hong et al. (2023) developed a qPCR method for detecting the endangered Asian giant softshell turtle, *Pelochelys cantorii*. The researchers found a significant linear relationship between turtle biomass and eDNA concentration in experimental pools and noted that eDNA concentration decreases over time once the species is removed from the water.

7. Picard et al. (2023) investigated the detection of two non-native fish species, European perch and rudd, in small shallow lakes in Aotearoa New Zealand using eDNA. Droplet digital PCR assays were applied to water and sediment samples from multiple sites within the lakes. The study recommended at least six sites and five replicates for sediment samples, and twelve sites with eight replicates for water samples to achieve reliable detection. The findings will help refine eDNA sampling strategies for monitoring and managing invasive fish species in freshwater ecosystems.

8. Quebedeaux et al. (2023) focused on the Boston Mountains Crayfish (*Cambarus causeyi*), a rare species in Arkansas, United States. While conventional methods detected the crayfish at only 17.6% of sites, the eDNA assay identified the species at 24.0% of sites. The findings suggested eDNA could play a crucial role in monitoring endangered burrowing crayfish.

9. Holmes et al. (2024) investigated the sensitivity of eDNA detection in turbid water, specifically targeting delta smelt, a critically endangered fish. Field sampling showed weak eDNA signals, prompting laboratory experiments with water spiked at different turbidity levels. The study evaluated different filtration methods and prefiltration steps, finding that glass fiber filters yielded the highest detection rates. Turbidity significantly affected eDNA detection, and interactions between filter types and prefiltration were important.

### Ecological health assessment and biomonitoring

10. Aunins et al. (2023) evaluated the use of eDNA for assessing arthropod diversity in streams comparing results with traditional surveys. The researchers sampled the Potomac River watershed (Maryland, United States), and observed correlations between eDNA-derived metrics and manual Benthic Invertebrate (BI) indices of environmental quality. Although taxa overlap was limited between methods, eDNA metrics effectively differentiated between high- and low-quality sites. The findings suggested that eDNA can be a rapid and informative alternative to traditional BI surveys for stream condition assessments.

11. Callac et al. (2023) examined the microbial dynamics in the rearing water of Pacific blue shrimp larvae, investigating how these microbes influence larval health and survival. Using 16S rRNA gene sequencing, the research identified specific microbial taxa, correlated with healthy larvae, and others linked to mortality. The results indicate microbial biomarkers could be used for early bio-surveillance to optimize rearing conditions and improve larval survival rates in shrimp aquaculture.

12. Coppo et al. (2023) investigated the impact of a mining disaster on meiofaunal assemblages in the Rio Doce estuary (Brazil), comparing eDNA metabarcoding data from 1.7 years and 2.8 years post-contamination. Results showed a shift in community composition, with Arthropoda dominating after 2.8 years. Despite a reduction in sediment metal concentrations, meiofaunal diversity remained affected, indicating that sediment contaminants continued to influence benthic assemblages.

13. Chavarria, Batista & Saltonstall (2024) evaluated microbial water quality in 21 streams in the Panama Canal Watershed, across four land uses (mature forest, secondary forest, silvopasture, and cattle pasture), comparing culture-based fecal coliform tests with 16S rDNA metabarcoding. While culturing detected coliforms and *E. coli* across all sites, metabarcoding revealed variability in coliform abundance, and identified genera causing false positives in culture tests.

14. Wilkinson et al. (2024) presented a new taxon-independent community index (TICI) for eDNA-based bioassessment of riverine health. TICI condenses eDNA metabarcoding data into an ecological health metric by assigning health indicator values to Amplicon Sequence Variants (ASVs). When applied to 53 river sites in Aotearoa New Zealand, TICI scores strongly correlated with traditional macroinvertebrate indices.

### Species detection

15. Feng & Lougheed (2023) developed species-specific eDNA protocols to detect the common musk turtle (*Sternotherus odoratus*), assessing its distribution near its northern range limit in Southern Ontario, Canada. The results indicated that traditional observations may underestimate the turtle’s distribution and highlighted the effectiveness of eDNA in understanding the distribution of cryptic aquatic species.

16. Gonzalez Colmenares et al. (2023) evaluated the use of eDNA for detecting fish spawning aggregations off Puerto Rico’s west coast. Despite developing primers and validating sample collection during peak spawning, species-specific detection failed due to low eDNA concentrations, contamination from non-target DNA, and environmental factors. The authors recommend improvements for future eDNA studies, particularly for monitoring fish populations in tropical regions.

17. Simpson et al. (2023) developed a species-specific qPCR assay to detect the invasive Asian paddle crab *Charybdis japonica*. The qPCR assay was highly specific to *C. japonica* and did not cross-amplify with related species. Its ability to detect the species from complex substrates shows promise for integration into marine biosecurity programs.

### Applications & management

18. Gold et al. (2022) produced a guide outlining five key steps for implementing eDNA metabarcoding in marine ecosystem monitoring. The steps identified were selecting target genes, developing reference databases, ensuring decontamination protocols, conducting pilot studies, and archiving data. A case study in the ports of Los Angeles and Long Beach (United States) showed eDNA detected 94.1% of species found in traditional surveys. An additional 55 native fish were detected using eDNA, highlighting the utility of this approach.

19. Leontidou, Rubel & Stoeck (2023) compared two approaches—quantile regression splines (QRS) and supervised machine learning (SML)—for assessing environmental quality at coastal marine aquaculture sites. Using bacterial eDNA metabarcoding data from aquaculture farms in Norway and Scotland, the study found both methods effective at predicting environmental quality (89–90% accuracy), with SML showing slightly higher accuracy. Both methods demonstrated congruence in identifying key amplicon sequence variants (ASVs) related to environmental health.

20. Shea et al. (2023) systematic review analyzed 60 marine eDNA metabarcoding studies to identify barriers to data usability and accessibility. Key challenges included inconsistent metadata, limited supplementary information, and a concentration of research in the US. However, authors showed consistency in data storage and a trend toward open access publishing. The study emphasized the need for universal guidelines to enhance the accessibility and use of eDNA data as the field continues to grow.

## Conclusion

The studies in this special issue highlight the potential for the use of eDNA based tools to improve aquatic monitoring and management. Three areas emerged as priorities for advancing the field of aquatic molecular monitoring.

(1) **Standardization.** This is essential for ensuring consistency and comparability across studies. Developing global standards for eDNA and eRNA sampling, analysis, and interpretation will facilitate collaboration and data integration, maximizing the impact of molecular tools on ecosystem management.

(2) **Integration with emerging technologies**. Combining molecular data with remote sensing, artificial intelligence, and ecological modelling will enhance our understanding of ecosystem dynamics and improve predictive capabilities. These interdisciplinary approaches will be critical for addressing complex environmental challenges and informing evidence-based management decisions.

(3) **Integration in decision making policy.** Engaging stakeholders, including policymakers, and industry representatives will bridge the gap between science and implementation. Equally important is ensuring the outputs of these tools are fit for purpose and can easily be used for decision making.

Notably, none of the articles in this special issue used eRNA, which has potential for exploring and differentiating living organisms from legacy DNA (Pochon et al., 2017; Cristescu, 2019). Future research should prioritize the development of eRNA-specific methodologies. The studies in this special issue highlight recent advancements in the field of molecular monitoring, and showcase the potential of using eDNA technologies to improve management. Interdisciplinary collaboration, technological innovation, and stakeholder/policy maker engagement will be key to unlocking the full potential of these rapidly developing approaches.

## Additional Information and Declarations

### Data Availability

The following information was supplied regarding data availability: This is an Editorial.
